# Development of Attractive Toxic Sugar Baits (ATSBs) System and Its Effectiveness in Mosquito Control

**DOI:** 10.3390/insects16030258

**Published:** 2025-03-02

**Authors:** Ruixiang Zhang, Teng Zhao, Dan Xing, Xinyu Zhou, Haotian Yu, Dongfen Geng, Zhihua Fan, Kai Wang, Xinan Huang, Chunxiao Li

**Affiliations:** 1 Artemisinin Research Center, Guangzhou University of Chinese Medicine, Guangzhou 510405, China; 2 State Key Laboratory of Pathogen and Biosecurity, Beijing 100071, China

**Keywords:** ATSB, attractants, insecticides, preservative, mosquito control

## Abstract

Background: Attractive Toxic Sugar Baits (ATSBs) are an innovative vector control strategy based on the “attract-and-kill” principle. The core of ATSBs lies in the preparation of attractive and toxic baits through the mixing and proportioning of luring and active ingredients. Although previous studies have investigated the effects of ATSBs on mosquitoes, significant challenges remain for broader field application. Methods: This study evaluated five fruit juices as ATSBs for mosquitoes, focusing on feeding preferences. Preservative concentrations were assessed by measuring antimicrobial activity over time. Two commercial traps were tested for mosquito entry rates. The optimal insecticide species and concentration were determined based on mortality rates. An optimized ATSBs system was developed and tested under a semi-field cage. Statistical analysis was performed using GraphPad Prism. Results: Within 24 h, apple juice-based ATSBs had the highest attractant index for *Culex quinquefasciatus* and *Anopheles sinensis*, while a pear juice-based ATSB was most effective for *Aedes albopictus*. A 0.1% preservative concentration best maintained juice stability. The LC50 values of dinotefuran-based ATSBs for *Cx. quinquefasciatus*, *Ae. albopictus*, and *An. sinensis* were 1.18 × 10^−3^, 4.06 × 10^−4^, and 5.20 × 10^−5^ g/L, respectively. The *Spodoptera frugiperda* trap outperformed the *Drosophilidae* trap. Simulated semi-field cage tests showed 48 h mortality rates of 86.00% for *Cx. quinquefasciatus* and 95.67% for *Ae. albopictus*. Conclusion: This study optimized an ATSB system by screening various fruit juices, preservative concentrations, insecticides, and trap devices. The system’s efficacy in mosquito control was evaluated under a semi-field cage. These findings provide a strong foundation for the future application and refinement of ATSB-based mosquito control strategies.

## Simple Summary

This study focuses on the development of a mosquito control system—Attractive Toxic Sugar Baits (ATSBs)—and its effectiveness in mosquito control. By systematically screening attractants, preservative concentrations, insecticides, and commercial traps, we have successfully integrated an ATSB system suitable for field applications. In semi-field cage experiments, the ATSB system demonstrated remarkable efficacy in killing mosquitoes, highlighting its potential as a sustainable tool for mosquito control in real-world environments. These findings provide a strong foundation for the future application and refinement of ATSB-based mosquito control strategies.

## 1. Introduction

Mosquitoes serve as significant vectors for a wide range of viral diseases, posing a serious threat to public health. These include dengue, yellow fever, Japanese encephalitis, West Nile fever, Zika virus, chikungunya, and Sindbis virus [1,2]. It is estimated that approximately 4 billion people worldwide are at risk of contracting mosquito-borne diseases, with tropical and subtropical regions experiencing the highest levels of disease transmission [3]. Although some mosquito-borne pathogens have long been known to humans and have posed persistent threats, recent years have witnessed the emergence of new pathogens, presenting unprecedented epidemiological risks and significant challenges to human health [4]. Current mosquito control strategies are inadequate for sustained disease mitigation, necessitating the development of effective and environmentally safe integrated vector control management approaches. Promising emerging techniques include the sterile insect technique (SIT), gene-driven methods, incompatible insect technique (IIT), release of insects carrying dominant lethal genes (RIDL), and Attractive Toxic Sugar Baits (ATSBs), all of which show significant potential for mosquito control [5].

Attractive Toxic Sugar Baits (ATSBs) are a novel integrated vector control strategy based on mosquitoes’ natural attraction to sugar sources. This method achieves population control by combining an attractant with a lethal insecticide, which entices mosquitoes to feed on the toxic sugar bait, ultimately resulting in their mortality [6]. ATSB solutions typically comprise an attractant preferred by the target insect, a sugar source, and an insecticide. This strategy fully leverages the behavioral characteristics of mosquitoes and can effectively reduce mosquito densities by deploying mixtures of toxins and sugar sources near mosquito habitats, such as larval breeding sites, or in areas like trees within the host’s resting environment [7,8,9,10].

In 1965, Lea [11] introduced the concept of Attractive Toxic Sugar Baits (ATSBs) in laboratory experiments. Based on mosquitoes’ natural behavior of feeding on sugar, researchers developed Toxic Sugar Baits (TSBs), incorporating the insecticide malathion into the bait to control mosquito populations. However, mosquitoes primarily feed on sugar to obtain carbohydrates necessary for their survival [12]. It was soon recognized that TSBs, due to their non-volatile nature, could not compete with natural sugar sources that contained abundant attractants [13]. This limitation led to the development of Attractive Toxic Sugar Baits (ATSBs). In nature, various attractants, such as flowers, nectar, and decaying fruit, are prevalent, and studies have shown that the scents of flowers, rotting fruit, honey, and synthetic fragrances strongly attract mosquitoes [14,15].

In addition to attractants, insecticidal components play a crucial role in the effectiveness of the ATSBs strategy. To date, Fiorenzano [16] have compiled several toxic compounds, including chemical insecticides, biopesticides, and plant-derived products. The chemical insecticides used in ATSB formulations include various classes approved for vector control, such as carbamates, organophosphates, phenylprazoles, pyrethroids, pyrroles, neonicotinoids, and macrocyclic lactones [17,18,19,20,21]. Other insecticidal agents, such as boric acid [22,23,24,25], eugenol [26,27], and additional chemicals, have also been investigated. Moreover, insecticides like fipronil [28,29,30], ivermectin [31,32,33], sodium ascorbate [34], deltamethrin [19,35,36], and microencapsulated garlic oil [37,38] have shown effective mosquito control in both laboratory and field trials.

The ATSBs mosquito control strategy aligns closely with the One Health philosophy, aiming to protect human, animal, and environmental health by reducing mosquito-borne diseases and enhancing global public health. As one of the five emerging mosquito control technologies recommended by the WHO, the ATSBs strategy has relatively mature products in Africa [39] but has yet to be introduced in China. There is an urgent need to develop ATSB formulations targeting the primary vector mosquito species in China. Therefore, we are committed to developing stable, convenient ATSB formulations tailored to the main vector mosquito species in China, with the aim of enhancing their adaptability and efficacy in domestic field environments. In this study, commercial fruit juice was used as an attractant to test its ability to lure mosquitoes. The effect of various preservative concentrations on mosquito feeding behavior under high temperature and high humidity conditions was evaluated, with the goal of achieving long-term stability and practicality for ATSB formulations. We screened highly effective chemical insecticides to enhance the efficacy of ATSB and evaluated trapping devices to improve their suitability for field operations. Ultimately, this study integrated an ATSBs system tailored for field environments and verified its mosquito-killing efficacy in a simulated field, providing both a theoretical foundation and practical basis for future application in field settings.

## 2. Materials and Methods

### 2.1. Mosquito Strains

The mosquito species used in this study included *Aedes albopictus* (Nanjing strain), *Culex quinquefasciatus* (Haikou strain), and *Anopheles sinensis* (Wuxi strain). All strains were long-term laboratory colonies maintained for several years by the state key laboratory of pathogens and biosecurity. Eggs of *Cx. quinquefasciatus* and *Ae. albopictus* were hatched and reared overnight in water, with larvae fed commercially available fish feed until pupation. In contrast, *An. Sinensis* eggs were hatched and reared in purified water, with larvae fed a mixture of pig liver powder, yeast powder, and rodent chow until pupation. After emergence, adult mosquitoes were maintained on an 8% sucrose solution and propagated using mouse blood for successive generations. The mosquitoes were reared under controlled conditions of 26 ± 1 °C, 70 ± 5% relative humidity, and a photoperiod of 14 h light and 10 h darkness.

### 2.2. Preparation of ATSB

#### 2.2.1. Preparation of Laboratory Attractants

Juice preparation: the fruit juice used in this study was sourced from Huiyuan (batch number: Q/MAAD0001S). The juice formulation consisted of concentrated fruit juice (corresponding fruit) and water, with no additional sugars or preservatives added. Commercial fruit juices (apple, grape, orange, pear, and peach) were each supplemented with 0.1% food dye (Sinopharm Chemical Reagent, Shanghai, China). The control group consisted of an 8% sucrose solution, to which an equal amount of food dye was added.

#### 2.2.2. Preparation of Laboratory Preservative Concentrations

Preservative: potassium sorbate (Macklin, Shanghai, China) was precisely weighed using a 0.01 g analytical balance, with quantities of 0.05 g, 0.1 g, and 0.5 g. These were then dissolved in 100 mL of apple juice to prepare preservative solutions with final concentrations of 0.05%, 0.1%, and 0.5%, respectively.

#### 2.2.3. Preparation of Laboratory Insecticides

Dinotefuran (Macklin, Shanghai, China): 0.1 g samples of dinotefuran were accurately weighed using a precision balance and then dissolved in 100 mL of purified water to prepare a 1 g/L stock solution. The stock solution was subsequently diluted with fruit juice to obtain various concentrations of dinotefuran–ATSB.

Boric acid (Sinopharm Chemical Reagent, Beijing, China): 0.1 g, 0.5 g, 1 g, and 2 g of boric acid were accurately weighed using a precision balance and then dissolved in fruit juice to prepare 0.1%, 0.5%, 1%, and 2% boric acid–ATSB solutions.

#### 2.2.4. Laboratory Trapping Device

**Figure 1:**
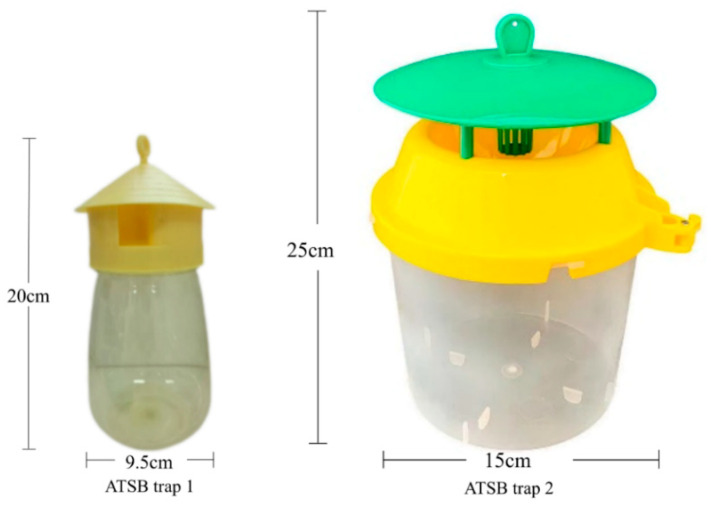
Photographic images of ATSB trap 1 and ATSB trap 2.

ATSB trap 1 is a commercially available *Drosophilidae* trap, measuring approximately 20 cm in height and 9.5 cm in width, purchased from the Gelvmei Horticultural Brand Store. ATSB trap 2 is a commercially available *Spodoptera frugiperda* trap, measuring approximately 25 cm in height and 15 cm in width, purchased from the Zhongjie Sifang Flagship Store. ATSB traps 1 and 2 were shown in Figure 1.

### 2.3. Laboratory ATSB Screening Method

#### 2.3.1. Laboratory ATSB Attractant Component Screening Method

Five experimental groups and five control groups were established, with mosquito cages appropriately labelled to correspond to each group. In the experimental groups, cotton balls were saturated with 25 mL of fruit juice, while in the control groups, cotton balls were saturated with an equal volume of the control solution. Within each mosquito cage, measuring 30 cm × 30 cm × 25 cm, cotton balls from the experimental and control groups were placed diagonally opposite each other to ensure equal accessibility [40]. The experimental mosquitoes were anesthetized using carbon dioxide, and 30 mosquitoes (15 males and 15 females), aged 3–10 days post-emergence and fasted for 12 h, were selected on ice and introduced into mosquito cages. The experiment was conducted under laboratory conditions with a temperature of 26 ± 1 °C and a relative humidity of 70 ± 5%. The procedure was repeated three times, and the mosquitoes were left undisturbed overnight. On the following day, the numbers of male and female mosquitoes that fed on the cotton balls from the experimental and control groups were recorded separately, and the attraction index (AI) was calculated. The criterion for determining mosquito baiting ATSB is to shine a torch on the mosquito’s thorax and abdomen and observe the presence of pigment color to determine the bait consumed.

$$Attraction\;index\;(AI)=\frac{Average\;mosquitoes\;feeding\;rate\;in\;the\;treatment\;group}{Average\;mosquitoes\;feeding\;rate\;in\;the\;control\;group}$$

#### 2.3.2. Laboratory ATSB Preservative Component Screening Method

Commercial apple juice supplemented with preservatives at varying concentrations was stored in a temperature-controlled incubator at 40 °C and 70–75% humidity for periods of seven and thirty days. After seven and thirty days, 0.1% red food dye was added to the juice for mosquito feeding. The control group consisted of fresh apple juice with an equal amount of red food dye. Mosquito cages were labelled according to the experimental groups. *Cx. Quinquefasciatus* mosquitos, anesthetized with carbon dioxide, were selected: 30 mosquitoes (15 males and 15 females), aged 3–10 days and fasted for 12 h, were introduced into the labelled cages. Cotton balls soaked in equal volumes of the prepared solutions were placed inside the cages [19]. The experiment was conducted under laboratory conditions of 26 ± 1 °C and 70 ± 5% humidity, repeated in triplicate. After 24 h, the number of mosquitoes that fed was counted, and the feeding rate was calculated.

#### 2.3.3. Laboratory ATSB Insecticidal Component and Concentration Screening Method

Mosquito cages were labelled according to the experimental groups, and the experiment was conducted in triplicate. The mosquitoes were anesthetized with carbon dioxide, selected on ice, and placed in separate 30 cm × 30 cm × 25 cm cages for screening the optimal concentrations of insecticides. Thirty mosquitoes (15 males and 15 females), aged 3–10 days and fasted for 12 h, were introduced into each cage. Cotton balls soaked in 25 mL of the experimental ATSB solution or 25 mL of 8% sucrose solution (control group) were placed in the cages. The laboratory conditions were maintained at 26 ± 1 °C and 70 ± 5% humidity. Mortality was observed at 24 and 48 h to determine the most effective insecticide and its optimal concentration, with LC50 (lethal concentration killing 50% of exposed mosquitoes) and LC90 (lethal concentration killing 90% of exposed mosquitoes) calculated as key efficacy metrics. The experiment was repeated three times for each group. The criteria for determining mosquito death are as follows: the abdomen is upturned, all six legs exhibit twitching, and the mosquito is unable to flip over or crawl when prodded with an insect needle [35].

#### 2.3.4. Laboratory ATSB Trap Screening Method

Mosquito cages were labelled according to their respective experimental groups. The experimental and control fruit juices were placed in petri dishes and positioned in ATSB traps 1 and 2, respectively, within a 50 cm × 50 cm × 50 cm mosquito cage. The control group contained fruit juice with preservatives and dyes, while the experimental group contained fruit juice with preservatives, dyes, and 0.01 g/L dinotefuran. Twenty *Cx. quinquefasciatus* mosquitoes (10 males and 10 females), aged 3–10 days and fasted for 12 h, were introduced into each cage. The laboratory conditions were maintained at 26 ± 1 °C and 70 ± 5% humidity, and the experiment was repeated three times. Every 24 h, the number of mosquitoes entering each trap was recorded to calculate the entry rate [40].

#### 2.3.5. Mortality Test of Experimental Mosquitoes in Semi-Field Cage with ATSB

**Figure 2:**
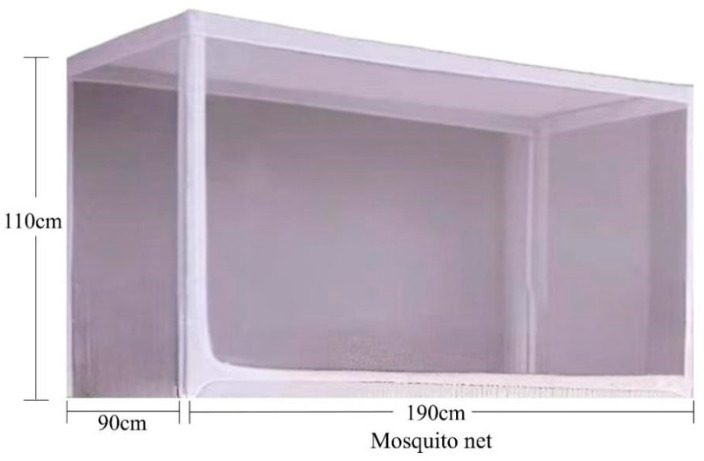
Photographic images of semi-field cage.

A mosquito net (90 cm × 110 cm × 190 cm) was used to simulate field conditions (Figure 2). The net was labelled according to the experimental groups. Mosquitoes were anesthetized with CO_2_, and 200 mosquitoes (100 males and 100 females), aged 3–10 days and fasted for 12 h, were selected and introduced into the mosquito net. For each group, 50 mL of either the experimental or control solution was absorbed by a 3 cm × 3 cm × 3 cm sponge and placed in the mosquito net. The experiment was repeated three times. The number of dead mosquitoes was recorded at 24 h and 48 h [41].

### 2.4. Statistical Analysis

Data were analyzed using GraphPad Prism (Version 8.3.0, GraphPad Software, San Diego, CA, USA). The normality of the data was tested using the Shapiro–Wilk test, and variance homogeneity was assessed using the Brown–Forsythe test. For two-group comparisons, a *t*-test was applied when the data were normally distributed with equal variances, while Welch’s *t*-test was used when variances were unequal. For non-normally distributed data, the non-parametric Mann–Whitney U test was employed. For comparisons among multiple groups, one-way analysis of variance (ANOVA) was used when the data were normally distributed with equal variances, and Brown–Forsythe and Welch’s ANOVA were used for unequal variances. For non-normally distributed data, the Kruskal–Wallis test followed by Dunn’s multiple comparison test was applied. A *p*-value of <0.05 was considered statistically significant.

## 3. Results

### 3.1. Screening of Attractants in the ATSB System

This study evaluated the attractiveness of different fruit juices to various mosquito species. As shown in Table 1, apple juice had the highest attraction index for *Cx. Quinquefasciatus* (AI = 2.23) and *An. sinensis* (AI = 2.09), while pear juice was the most attractive to *Ae. albopictus* (AI = 1.12). For male *Cx. quinquefasciatus*, apple juice significantly outperformed orange juice (62.22% vs. 22.22%, *p* = 0.01) and peach juice (62.22% vs. 31.11%, *p* = 0.0438). A significant difference was also observed between orange juice and pear juice (22.22% vs. 53.33%, *p* = 0.0438). These results suggested that apple juice was the most attractive to male *Cx. quinquefasciatus*, while orange juice was the least effective attractant.

As shown in Table 1, for *Ae. albopictus* females, significant differences were observed between apple juice and grape juice (42.22% vs. 22.22%, *p* = 0.039), apple juice and orange juice (42.22% vs. 20.00%, *p* = 0.0215), grape juice and pear juice (22.22% vs. 46.67%, *p* = 0.0120), and orange juice and pear juice (20.00% vs. 46.67%, *p* = 0.0068). These results suggested that pear juice was more attractive to *Ae. albopictus* females than grape juice or orange juice, with orange juice being the least effective. Therefore, apple juice was identified as the most effective attractant for *Cx. Quinquefasciatus* (AI = 2.23) and *An. sinensis* (AI = 2.09), while pear juice was the most effective for *Ae. albopictus* (AI = 1.12).

### 3.2. Screening of Preservative Concentration in the ATSBs System

**Figure 3:**
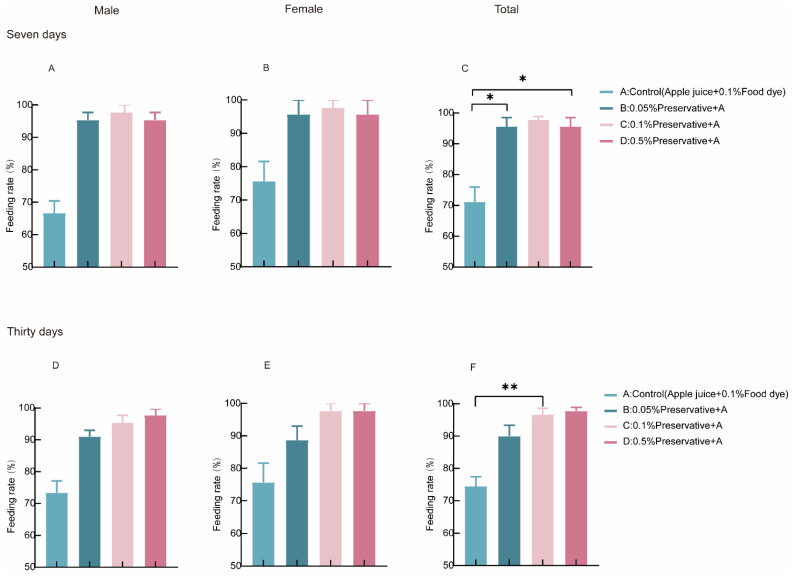
Feeding rates of *Cx. quinquefasciatus* on fruit juice stored for seven and thirty days at different preservative concentrations. The apple juice samples containing 0.05%, 0.1%, and 0.5% preservatives were maintained under controlled conditions at 40 °C and 70% relative humidity for 7 and 30 days. (**A**) The 24 h feeding rate of male *Cx. quinquefasciatus* on 7-day-stored apple juice. (**B**) 24 h feeding rate of female *Cx. quinquefasciatus* on 7-day-stored apple juice. (**C**) The 24 h feeding rate of total *Cx. quinquefasciatus* on 7-day-stored apple juice. (**D**) The 24 h feeding rate of male *Cx. quinquefasciatus* on 30-day-stored apple juice. (**E**) The 24 h feeding rate of female *Cx. quinquefasciatus* on 30-day-stored apple juice. (**F**) The 24 h feeding rate of total *Cx. quinquefasciatus* on 30-day-stored apple juice. * *p* < 0.05, ** *p* < 0.01.

This study evaluated the effectiveness of different preservative concentrations on fruit juice preservation. After each period, mosquitoes were exposed to the juice, and their feeding rates were recorded after 24 h. As shown in Figure 3A–C, after one week, the feeding rates of male, female, and total *Cx. quinquefasciatus* were significantly higher in the preservative-treated groups compared to the control group.

After one month (Figure 3D–F), the feeding rates of male, female, and total mosquitoes remained significantly higher in the preservative-treated groups compared to the control. In Figure 3C, the 0.05% preservative group showed a significant difference from the control group (71.11% vs. 95.56%, *p* = 0.0125), as did the 0.5% preservative group (71.11% vs. 95.56%, *p* = 0.0125). Similarly, in Figure 3F, the 0.1% preservative group also showed a significant difference from the control group (71.11% vs. 95.56%, *p* = 0.0125), as did the 0.5% preservative group (74.44% vs. 96.67%, *p* = 0.0032). These results indicate that after one month, mosquitoes prefer the juice with a 0.1% preservative concentration in the meal. Thus, 0.1% was selected as the optimal concentration for subsequent experiments.

### 3.3. Screening of Insecticide Types and Concentrations in the ATSBs System

This study evaluated the effectiveness of insecticides at varying concentrations in feeding experiments with mosquitoes, recording mortality every 24 h. As shown in Table 2, the LC50 values of dinotefuran–ATSB for *Cx. quinquefasciatus*, *Ae. albopictus*, and *An. sinensis* were 1.18 × 10^−3^, 4.06 × 10^−4^, and 5.20 × 10^−5^ g/L, respectively. These results indicated that dinotefuran was most effective against *An. sinensis*, followed by *Ae. albopictus*. In comparison, the LC50 values of boric acid–ATSB for *Cx. quinquefasciatus*, *Ae. albopictus*, and *An. sinensis* were 15.05, 7.24, and 2.97 g/L, respectively, with boric acid being most effective against *An. sinensis*, followed by *Ae. albopictus*.

The LC50 values of dinotefuran–ATSB for male *Cx. quinquefasciatus*, *Ae. albopictus*, and *An. sinensis* were 8.13 × 10^−4^, 3.72 × 10^−4^, and 3.20 × 10^−5^ g/L, respectively, while for females, the LC50 values were 1.71 × 10^−3^, 4.45 × 10^−4^, and 8.40 × 10^−5^ g/L. Dinotefuran–ATSB exhibited higher mortality rates in males than in females across all mosquito species. For boric acid–ATSB, the LC50 values for males of *Cx. quinquefasciatus*, *Ae. albopictus*, and *An. sinensis* were 12.22, 5.02, and 2.72 g/L, respectively, while for females, the LC50 values were 21.39, 11.49, and 3.31 g/L. Similar to dinotefuran, boric acid–ATSB showed higher efficacy against males than females. Overall, dinotefuran–ATSB demonstrated superior insecticidal efficacy compared to boric acid–ATSB, making it the preferred insecticide for the ATSB system.

### 3.4. Screening of Trap Devices in the ATSBs System

**Figure 4:**
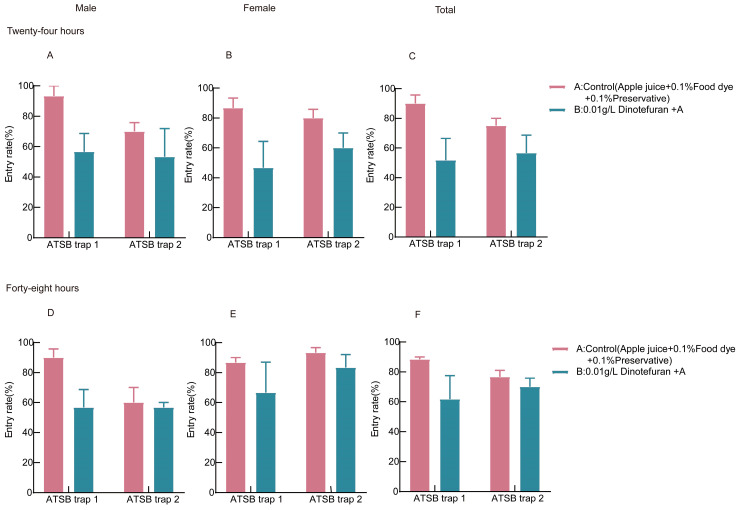
Entry rates of *Cx. quinquefasciatus* into different trap devices: (**A**) shows the 24 h entry rates of male *Cx. quinquefasciatus* into ATSB traps 1 and 2; (**B**) displays the 24 h entry rates of female *Cx. quinquefasciatus* into ATSB traps 1 and 2; (**C**) presents the 24 h entry rates of the total *Cx. quinquefasciatus* population into ATSB traps 1 and 2. Similarly, (**D**) illustrates the 48 h entry rates of male *Cx. quinquefasciatus* into ATSB traps 1 and 2; (**E**) depicts the 48 h entry rates of female *Cx. quinquefasciatus* into ATSB traps 1 and 2; and (**F**) shows the 48 h entry rates of the total *Cx. quinquefasciatus* population into ATSB traps 1 and 2.

Based on the results from Section 3.1, Section 3.2 and Section 3.3, the optimal attractants (apple juices), preservative concentration (0.1%), and insecticide (0.01 g/L dinotefuran) were selected to formulate the most effective ATSB mixture. Commercial trap devices 1 and 2 were used to evaluate the entry rate of *Cx. quinquefasciatus*. As shown in Figure 4A–C, in the control group, the average entry rates of male, female, and total mosquitoes into ATSB trap 1 ranged from 86.67% to 93.33%, while in ATSB trap 2, the entry rates ranged from 70.00% to 80.00%. This indicates that ATSB trap 2 had a lower entry rate than ATSB trap 1. In the experimental group, the average entry rates of male, female, and total mosquitoes into ATSB trap 1 ranged from 46.67% to 51.67%, while in ATSB trap 2, the entry rates ranged from 53.33% to 60.00%. These results suggest that ATSB trap 2 had a higher entry rate than ATSB trap 1 in the experimental group within 24 h.

Figure 4D–F shows that within 48 h, the average entry rates of male, female, and total *Cx. quinquefasciatus* mosquitoes in the control group were 86.67–90.00% for ATSB trap 1 and 60.00–93.33% for ATSB trap 2. In the experimental group, the entry rates ranged from 56.67% to 66.67% for ATSB trap 1 and 56.67% to 83.33% for ATSB trap 2. While the control group exhibited similar entry rates for both devices, ATSB trap 2 displayed higher entry rates in the experimental group. Overall, ATSB trap 2 demonstrated superior performance and was selected as the optimal ATSB trap device.

### 3.5. Insecticidal Efficacy of the ATSB System Against Culex quinquefasciatus and Aedes albopictus in Semi-Field Cage

Based on the findings from Section 3.1, Section 3.2, Section 3.3 and Section 3.4, the optimal attractants (apple juice and pear juice), preservative concentration (0.1%), insecticide and its concentration (0.01 and 0.1 g/L dinotefuran), and trap device (ATSB trap 2) were selected.

**Figure 5:**
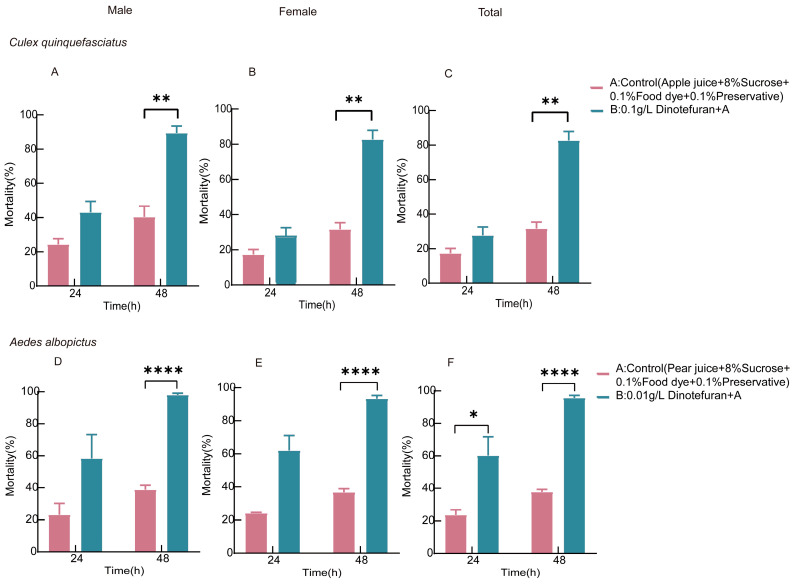
Insecticidal effect of ATSB on *Cx. quinquefasciatus* and *Aedes albopictus* in semi-field cage: (**A**) shows the 48 h mortality rate of male *Cx. quinquefasciatus* exposed to ATSB in semi-field conditions; (**B**) displays the 48 h mortality rate of female *Cx. quinquefasciatus* exposed to ATSB; and (**C**) presents the 48 h mortality rate of the total *Cx. quinquefasciatus* population. Similarly, (**D**) illustrates the 48 h mortality rate of male *Ae. albopictus* exposed to ATSB; (**E**) depicts the 48 h mortality rate of female *Ae. albopictus* exposed to ATSB; and (**F**) shows the 48 h mortality rate of the total *Ae. albopictus* population. * *p* < 0.05, ** *p* < 0.01, **** *p* < 0.0001.

This study assessed the efficacy of the optimized ATSBs system in controlling *Cx. quinquefasciatus* and *Ae. albopictus* under semi-field cage. Mosquitoes were housed in a mosquito net (90 cm × 110 cm × 190 cm), and mortality was recorded every 24 h. As shown in Figure 5A,B, the mortality rate of *Cx. quinquefasciatus* males in the experimental group was significantly lower than that in the control group within 48 h (40.33% vs. 89.33%, *p* = 0.003). Similarly, a significant difference was observed in the mortality rate of *Cx. quinquefasciatus* females between the experimental and control groups (31.67% vs. 82.67%, *p* = 0.0014). The data demonstrate that 0.1 g/L dinotefuran exhibited a higher efficacy in eliminating male *Cx. quinquefasciatus* compared to females.

As shown in Figure 5D,E, a significant difference in mortality rates was observed between the experimental and control groups for both male (38.67% vs. 98.00%, *p* < 0.0001) and female (36.67% vs. 93.33%, *p* < 0.0001) *Ae. albopictus* within 48 h. The data indicate that dinotefuran exerted a similarly significant lethal effect on both male and female *Ae. albopictus*, which was more pronounced than its effect on male and female *Cx. quinquefasciatus* (Figure 4A,B).

In Figure 5C,F, a significant difference in *Cx. quinquefasciatus* mortality was observed between the experimental and control groups within 48 h (36.00% vs. 86.00%, *p* = 0.0019). A significant difference in the mortality rate of *Ae. albopictus* was observed between the experimental and control groups at both 24 h (23.50% vs. 60.17%, *p* = 0.0382) and 48 h (37.67% vs. 95.67%, *p* < 0.0001). The results clearly indicate that dinotefuran is more effective in controlling *Ae. albopictus* than *Cx. quinquefasciatus*.

## 4. Discussion

As one of the five emerging mosquito control technologies recommended by the WHO, the ATSBs strategy has relatively mature products in Africa [39]. However, there is a need for more localized strategies to develop ATSB formulations tailored to the main vector species in China. In this study, a variety of commercially available fruit juices were evaluated for their attractiveness to target insects, as well as the concentrations of preservatives and insecticides they contained. Additionally, this study focused on identifying more effective trapping devices. Ultimately, a more stable and convenient ATSB formulation was developed to improve its adaptability and efficacy under field conditions. Locally available fresh or decaying fruit, as well as commercial fruit juices, were commonly employed as attractants in ATSB solutions. To minimize experimental bias, we used standardized commercial fruit juices and observed varying preferences among different mosquito species. For instance, apple juice exhibited the highest attraction index for *Cx. quinquefasciatus* and *An. sinensis* within 24 h (AI = 2.23, AI = 2.09), while pear juice showed the most pronounced attraction for *Ae. albopictus* (AI = 1.12). Several studies have indicated that guava juice is effective in attracting *Anopheles gambiae* and *Ae. albopictus*. For instance, Müller applied guava juice [7] fermented for 48 h, combined with 1% boric acid, to plants in Mali. After one week, the population of *An. gambiae* was reduced by approximately 90%. In Florida, Naranjo developed an ATSB formulation using locally available guava juice [42] fermented for 24 h with 3% boric acid. This formulation led to a 58% reduction in the *Ae. albopictus* population after seven days of field application.

Research indicates that volatile organic compounds (VOCs) in fruit juices, such as acetic acid and ethanol, exhibit significant attractiveness to mosquitoes, while their sugar content promotes feeding behavior by activating mosquito gustatory receptors [43]. Mosquitoes demonstrate sensitivity to specific colors, particularly darker shades, which may facilitate host localization. Environmental factors, including CO_2_ concentration, temperature, and humidity, significantly influence mosquito feeding and oviposition behaviors, with trace CO_2_ released during juice fermentation potentially enhancing bait efficacy [44]. Given the competition from multiple natural sugar sources in the environment, effective baits must possess sufficient attractiveness to outweigh the influence of these natural alternatives [14,43,44,45]. The attractiveness towards various fruits, along with the efficacy of their combination with insecticides, offers diverse options for mosquito control. While the use of single-brand commercial formulations may limit the ability to resolve the effects of specific ingredients (e.g., variations in apples from different origins), the primary components—apple juice concentrate and water—are carefully processed to ensure consistency [16]. And the standardized nature of commercial juices ensures the reproducibility of experimental materials, while their accessibility facilitates subsequent field applications [46]. Future research should further investigate variations in the attractant properties of commercial fruit products from different brands and species and their constituents across mosquito species. This could provide valuable insights into the ecological adaptability and sustainability of mosquito control strategies.

Regarding the use of preservatives, some researchers have utilized BaitStab™—a product developed by Westham Innovations LTD, which contains a slow-release agent and preservative. In field trials conducted in Israel and the United States, this product, mixed with 1% boric acid at working concentrations ranging from 1% to 10%, demonstrated over 95% mortality of *Anopheles sergentii* mosquitoes after 47 days, and 85% mortality of *Cx. quinquefasciatus* mosquitoes after 7 days [47,48]. This experiment selected potassium sorbate, a widely used food preservative, and tested it at various concentrations. Potassium sorbate effectively inhibits microbial growth, extending the shelf life of fruit juices at optimal levels [49,50]. Compared to alternatives like sodium benzoate, it offers superior safety, broader applicability, and minimal impact on flavor and color [51,52]. This study systematically evaluates the efficacy of ATSB formulations across potassium sorbate concentration gradients, providing an empirical basis for optimizing their ecological sustainability in field applications. This research focuses on *Cx. quinquefasciatus*, the most prevalent mosquito species in southern China’s urban ecosystems, as the standard test species due to its significantly higher population density compared to other species, as evidenced by monitoring data [53]. The selection is further justified by its role as the primary vector of Japanese encephalitis virus (JEV) [54], a major insect-borne disease in China, making its control crucial for regional public health safety. These results indicated that the attractiveness of the juice to *Cx. quinquefasciatus* remained significant even after one month at a preservative concentration of 0.1%. Taken together, the concentration of preservatives influences the effectiveness of ATSB. Consequently, the formulation was further optimized to enhance its efficacy and persistence, while minimizing the amounts of preservatives used to improve environmental sustainability and reduce ecological impact.

The optimized formulation was employed to screen insecticide concentrations for multiple mosquito species, taking into account the variations in insecticide sensitivity among different species [9]. Specifically, this experiment focused on evaluating the lethal effects of two commonly used insecticides, boric acid and dinotefuran, against three mosquito species: *Cx. quinquefasciatus*, *Ae. albopictus*, and *An. sinensis*. The results indicated that dinotefuran, at a concentration of 0.01 g/L, significantly increased mosquito mortality. Additionally, 2% boric acid achieved a 100% mortality rate for all three mosquito species within 48 h. Most studies have evaluated its effectiveness in killing mosquitoes using boric acid solutions ranging from 0.5% to 4%. For instance, Stewart (Tanzania) [20] formulated ATSB with 2% boric acid and tested it as a field bait station. After 16 days of testing, the mortality rates of *An. gambiae*, *Anopheles arabiensis*, and *Cx. quinquefasciatus* ranged from 36% to 48%, respectively. Additionally, the use of dinotefuran in ATSB has demonstrated significant lethal effects. At a concentration of 0.11%, dinotefuran can reduce *An. gambiae* densities by approximately 90% in the Mali region [21]. When the concentration is increased to 0.5%, it is capable of achieving nearly 100% mortality in both male and female populations of resistant *Cx. quinquefasciatus* within 24 h [35].

To prevent the misuse of chemical insecticides, researchers are striving to develop alternatives that are both effective and safe. Consequently, bioinsecticide and novel insecticides, including spinosad, *Bacillus thuringiensis*, and dinotefuran, are increasingly becoming focal points of research. Kumar employed ivermectin as an insecticide to develop ATSB in the Jordan Valley [32]. The experimental results indicated that the LC90 of ivermectin against *Anopheles culicifacies* and *Anopheles stephensi* were 19.8 ppm and 35 ppm, respectively. This study employed boric acid, which has been tested as a safe and environmentally friendly insecticide for vector-control [55], and dinotefuran (a neonicotinoid insecticide), classified as a WHO Category III slightly hazardous pesticide [56]. While demonstrating low mammalian toxicity, these compounds may still pose environmental risks through ATSB-mediated attraction of non-target species, particularly pollinators. Substantial evidence indicates that neonicotinoids can disrupt bee navigation [57], highlighting potential ecological impacts that warrant careful consideration. To advance ecological safety within the One Health framework, future research should integrate advanced toxicity assessment methods, including zebrafish embryo tests [58], with innovative delivery systems. Specifically, β-cyclodextrin-complexed garlic oil [37] and chitosan-based nanopesticides [59] show promise for enhancing target specificity and synergistic efficacy with botanical compounds [60], thereby facilitating the development of sustainable integrated pest management (IPM) systems [61]. Furthermore, establishing comprehensive monitoring protocols for non-target species exposure and implementing regional risk mitigation strategies are crucial for maintaining the equilibrium between effective vector control and ecosystem preservation.

This study evaluated two commercially available trapping devices, whose overall design effectively mitigated, to some extent, the impacts of sunlight and rainfall on the ATSB solution. The experimental results indicated that the difference in mosquito entry rates between the two devices was not statistically significant. However, the entry rate in the experimental group for device 2 (56.67–83.33%) was slightly higher than that for device 1 (56.67–66.67%). In less developed regions, ATSB traps are typically simple, handmade devices crafted by researchers. For instance, in a study conducted in Mali [62], traps were constructed using 1.5 L plastic beverage bottles with 2 cm openings and cotton threads inserted. The results showed that the mortality rate of *An. gambiae* exceeded 90% after 59 days of experimentation. Another study [30] conducted in an experimental shed in Côte d’Ivoire utilized a trapping device consisting of cotton pads soaked in 250 mL of ATSB solution. The pads were wrapped around a frame and suspended in a reservoir constructed from halved plastic water bottles, with consistent solution absorption maintained through cotton threads. This setup achieved a mortality rate of 19–39% for *An. gambiae* after an overnight exposure period. The ATSB system leverages commercially available trapping devices and juices, offering cost-effectiveness and accessibility, particularly in industrialized regions. These trapping devices demonstrate advantages in protection capability and operational ease, aligning with the core principle of ATSB technology—enabling rapid screening of effective attractants for immediate field application while supporting large-scale deployment in resource-constrained environments [21,62]. Future optimizations should focus on enhancing mosquito attraction, trapping efficiency, and stability under extreme weather conditions, while further reducing costs to improve adaptability.

This study conducted an initial evaluation of the optimized ATSB system in a semi-field cage. The experimental results demonstrated that 0.1 g/L dinotefuran achieved an 86.00% mortality rate against *Cx. quinquefasciatus* within 48 h, while 0.01 g/L dinotefuran resulted in a significantly higher mortality rate of 95.67% against *Ae. albopictus*, indicating greater sensitivity of *Ae. albopictus* to dinotefuran. The system demonstrated a significant lethal effect against *Cx. quinquefasciatus* and *Ae. albopictus* in the semi-field cage (*p* < 0.05), highlighting its potential for controlling target mosquito populations. This aligns with contemporary vector management strategies, which demonstrate a paradigm shift toward biological control dominance, supported by chemical assistance and advancements in genetic engineering. While gene drive systems and RIDL offer sustainable solutions with potential ecological implications, and SIT and IIT provide flexible, medium-term intervention capabilities despite their intensive production requirements, ATSB technology represents an advanced iteration of chemical control. ATSB maintains unique advantages in environmental compatibility and emergency response applications [5,63,64]. Future vector control strategies should integrate complementary technologies (e.g., SIT-IIT-ATSB combinations) with intelligent monitoring systems to achieve precision management. Concurrent implementation of robust public engagement and ethical oversight mechanisms will facilitate effective technology deployment.

## 5. Conclusions

This study optimized the ATSB system by selecting suitable attractants, preservative concentrations, insecticides, and trap devices. The system demonstrated significant mosquito mortality in semi-field cage tests, underscoring its potential for effective application in real-world mosquito control programs.

## Figures and Tables

**Table 1:** Attractiveness of different fruit juices to *Culex quinquefasciatus*, *Aedes albopictus*, and *Anopheles sinensis*.

		Apple Juice	Grape Juice	Orange Juice	Pear Juice	Peach Juice
*Culex quinquefasciatus*						
Average mosquito feeding rate ± SEM	Male	62.22 ± 5.88 ^ab^	51.11 ± 5.88	22.22 ± 8.01 ^ac^	53.33 ± 3.85 ^c^	31.11 ± 8.01 ^b^
	Female	46.67 ± 11.55	48.89 ± 9.69	33.33 ± 17.64	31.11 ± 18.19	40.00 ± 10.18
	Total	54.44 ± 5.56	50.00 ± 6.94	27.78 ± 5.88	42.22 ± 7.29	35.56 ± 6.19
Control	Male	15.56 ± 5.88	22.22 ± 8.89	31.11 ± 15.56	13.33 ± 7.70	15.56 ± 2.22
	Female	33.33 ± 10.18	28.89 ± 4.44	37.78 ± 11.76	53.33 ± 19.25	24.44 ± 5.88
	Total	24.44 ± 7.78	25.56 ± 5.88	34.44 ± 12.81	33.33 ± 12.02	20.00 ± 1.92
Attraction index	Male	4.00	2.30	0.71	4.00	2.00
	Female	1.40	1.69	0.88	0.58	1.64
	Total	2.23	1.96	0.81	1.27	1.78
*Aedes albopictus*						
Average mosquito feeding rate ± SEM	Male	31.11 ± 8.01	31.11 ± 5.88	22.22 ± 4.44	35.56 ± 2.22	28.89 ± 5.88
	Female	42.22 ± 5.88 ^ad^	22.22 ± 2.22 ^de^	20.00 ± 3.85 ^ac^	46.67 ± 3.85 ^ce^	33.33 ± 3.85
	Total	36.67 ± 1.92	26.67 ± 3.33	21.11 ± 2.94	41.11 ± 1.11	31.11 ± 4.44
Control	Male	37.78 ± 2.22	44.44 ± 5.88	42.22 ± 4.44	35.56 ± 5.88	28.89 ± 8.01
	Female	42.22 ± 2.22	33.33 ± 7.70	44.44 ± 8.01	37.78 ± 5.88	46.67 ± 7.70
	Total	40.00 ± 1.92	38.89 ± 2.94	43.33 ± 1.92	36.67 ± 5.09	37.77 ± 7.78
Attraction index	Male	0.82	0.70	0.53	1.00	1.00
	Female	1.00	0.67	0.45	1.24	0.71
	Total	0.92	0.69	0.49	1.12	0.82
*Anopheles sinensis*						
Average mosquito feeding rate ± SEM	Male	48.89 ± 5.88	37.78 ± 8.01	33.33 ± 3.85	24.44 ± 4.44	28.89 ± 5.88
	Female	57.78 ± 5.883	53.33 ± 3.85	40.00 ± 13.88	37.78 ± 14.57	37.78 ± 11.11
	Total	53.33 ± 3.85	45.56 ± 5.88	36.67 ± 7.70	31.11 ± 6.76	33.33 ± 5.77
Control	Male	22.22 ± 8.89	28.89 ± 5.88	33.33 ± 10.18	33.33 ± 13.88	20.00 ± 6.67
	Female	28.89 ± 9.69	31.11 ± 5.88	44.44 ± 5.88	31.11 ± 9.69	42.22 ± 9.69
	Total	25.56 ± 9.09	30.00 ± 0.00	38.89 ± 7.29	32.22 ± 11.60	31.11 ± 5.56
Attraction index	Male	2.20	1.31	1.00	0.73	1.44
	Female	2.00	1.71	0.90	1.21	0.89
	Total	2.09	1.52	0.94	0.97	1.07

Note: The experiment was repeated three times, with each repetition consisting of *n* = 30 (15 males and 15 females), for a total of *n* = 90. Means within the same row followed by the same letter were significantly different (*p* < 0.05). The letters represent the following comparisons: (^a^) apple vs. orange (*p* < 0.05), (^b^) apple vs. peach (*p* < 0.05), (^c^) orange vs. pear (*p* < 0.05), (^d^) apple vs. grape, and (^e^) grape vs. pear (*p* < 0.05), as determined by the Dunn test.

**Table 2:** Toxicity of dinotefuran and boric acid baits to male and female *Culex quinquefasciatus*, *Aedes albopictus*, and *Anopheles sinensis*, in the laboratory after 24 h exposure.

Species and Sex	LC50 (95%CI ^a^) g/L	LC90 (95%CI ^a^) g/L
*Culex quinquefasciatus*		
Total		
Dinotefuran	1.18 × 10^−3^	1.03 × 10^−2^
Boric acid	15.05 (13.52–16.90)	29.70 (24.50–41.74)
Male		
Dinotefuran	8.13 × 10^−4^ (5.00 × 10^−4^–1.30 × 10^−3^)	8.09 × 10^−3^ (4.34 × 10^−3^–2.16 × 10^−2^)
Boric acid	12.22 (11.02–13.56)	17.71 (15.61–21.85)
Female		
Dinotefuran	1.71 × 10^−3^	1.17 × 10^−2^
Boric acid	21.39 (16.80–45.38)	61.14 (34.18–80.11)
*Aedes albopictus*		
Total		
Dinotefuran	4.06 × 10^−4^	9.57 × 10^−4^
Boric acid	7.24	30.47
male		
Dinotefuran	3.72 × 10^−4^ (2.94 × 10^−4^–4.54 × 10^−4^)	9.17 × 10^−4^ (7.25 × 10^−4^–1.29 × 10^−3^)
Boric acid	5.02	24.04
Female		
Dinotefuran	4.45 × 10^−4^	9.76 × 10^−4^
Boric acid	11.49 (9.11–13.42)	23.17 (18.62–38.53)
*Anopheles sinensis*		
Total		
Dinotefuran	5.20 × 10^−5^	3.26 × 10^−4^
Boric acid	2.97	6.99
Male		
Dinotefuran	3.20 × 10^−5^ (1.70 × 10^−5^–5.60 × 10^−5^)	2.66 × 10^−4^ (1.34 × 10^−4^–9.53 × 10^−4^)
Boric acid	2.72 (2.02–3.54)	6.87 (5.13–10.64)
Female		
Dinotefuran	8.40 × 10^−5^ (5.40 × 10^−5^–1.32 × 10^−4^)	3.26 × 10^−4^ (1.93 × 10^−4^–8.72 × 10^−4^)
Boric acid	3.31 (2.45–4.17)	7.03 (5.49–10.17)

^a^ 95%CI, 95% lower confidence limit, and 95% upper confidence limit.

## Data Availability

The original contributions presented in this study are included in this article. Further inquiries can be directed to the corresponding authors.

## Abbreviations

The following abbreviations are used in this manuscript:

ATSBs	Attractive Toxic Sugar Baits
AI	Attraction index
SIT	Sterile Insect Technique
IIT	Incompatible Insect Technique
RIDL	Release of Insects carrying Dominant Lethal genes
